# Rivaroxaban treatment discontinuation rates in patients with nonvalvular atrial fibrillation in Italian clinical practice: RITMUS-AF

**DOI:** 10.1371/journal.pone.0341633

**Published:** 2026-02-12

**Authors:** Carmine Pizzi, Leopoldo Pagliani, Luca Dalle Carbonare, Piergiuseppe Agostoni, Egidio Imbalzano, Gennaro Galasso, Gianni Casella, Nicolò Dasseni, Leonardo De Luca, Italo Porto, Biagio Sassone, Davide Tosarello, Pareen Vora, Claudia Erminero, Giovanni Battista Forleo, Paolo Fusco, Ciro Mauro, Francesco Milone, Gian Piero Perna

**Affiliations:** 1 Cardiology Unit, IRCCS Azienda Ospedaliera-Universitaria di Bologna, Bologna, Italy; 2 Department of Medical and Surgical Sciences - DIMEC - Alma Mater Studiorum, University of Bologna, Bologna, Italy; 3 Veneto Institute of Oncology I.O.V. - IRCCS, Padua, Italy; 4 Department of Engineering for Innovation Medicine, University of Verona, Verona, Italy; 5 Centro Cardiologico Monzino IRCCS, Milan, Italy; 6 Department of Clinical and Community Sciences, University of Milan, Milan, Italy; 7 Department of Clinical and Experimental Medicine, Polyclinic University of Messina, Messina, Italy; 8 Department of Medicine, Surgery and Dentistry, University of Salerno, Baronissi, Italy; 9 Division of Cardiology, Ospedale Maggiore, Azienda AUSL di Bologna, Bologna, Italy; 10 Azienda Socio-Sanitaria Territoriale della Franciacorta, Ospedale di Chiari, Chiari, Italy; 11 Division of Cardiology, Fondazione IRCCS Policlinico San Matteo, Pavia, Italy; 12 IRCCS Azienda Ospedaliera Universitaria San Martino, Genova, Italy; 13 Cardiothoracic Vascular Department, Division of Provincial Cardiology, University of Ferrara, Ferrara, Italy; 14 Bayer S.p.A., Milan, Italy; 15 Bayer AG, Berlin, Germany; 16 ASST Fatebenefratelli Sacco, Milan, Italy; 17 Ambulatorio Specialistico Internistico Geriatrico, distretto 2 Latina, Latina, Italy; 18 Azienda Ospedaliera di Rilievo Nazionale Antonio Cardarelli, Naples, Italy; 19 Humanitas Gradenigo, Turin, Italy; 20 Azienda Ospedaliero Universitaria delle Marche, Ancona, Italy; Oswaldo Cruz Foundation, BRAZIL

## Abstract

Nonadherence to direct oral anticoagulant (DOAC) therapy exposes patients with nonvalvular atrial fibrillation (NVAF) to an increased risk of ischemic stroke and systemic embolism. Nevertheless, approximately 20% of patients discontinue treatment within the first year. In Italy, data on DOAC discontinuation rates are limited, especially in high-risk populations. RITMUS-AF, a prospective, observational cohort study conducted in 31 centers across Italy, investigated rivaroxaban treatment discontinuation in patients with NVAF in routine clinical practice. It included 805 patients aged ≥18 years with NVAF who were newly initiated on rivaroxaban. The primary endpoint was the proportion of patients who discontinued treatment during a 24-month follow-up. Secondary endpoints included the reasons for discontinuation and self-reported adherence to rivaroxaban therapy. At baseline, most patients were oral anticoagulant (OAC)-naïve (n = 599, 74.4%) and had either symptomatic (n = 364, 45.2%) or asymptomatic (n = 441, 54.8%) NVAF. The overall rate of rivaroxaban discontinuation was 8.9 per 100 patient-years (95% CI: 7.1–11.0). The main reasons for discontinuation were adverse events or physician decisions. After 24 months, high adherence was reported in 90.9% of OAC-non-naïve patients and 61.5% of OAC-naïve patients. Forty-six patients (5.7%) experienced bleeding events (with major bleeding events occurring in <0.5% of cases), and one patient (0.1%) had an ischemic stroke. In the RITMUS-AF study, rivaroxaban treatment was associated with a low treatment discontinuation rate, along with high self-reported adherence and a relatively low incidence of ischemic stroke and bleeding events in a high-risk population, findings that may help inform clinical decision-making on the use of rivaroxaban in routine practice.

## Introduction

Atrial fibrillation (AF) is associated with an increased risk of stroke and mortality, particularly among older individuals [1–7]. Direct oral anticoagulants (DOACs) substantially reduce stroke risk in patients with AF [7–11], but treatment patterns can vary depending on individual characteristics, including age, comorbidities and functional disabilities, and prior use of anticoagulants. Given these variations in patient characteristics, understanding and maintaining adherence is essential. Adherence to therapy is a critical determinant of outcomes in chronic diseases [12]; however, multiple factors influence medication-taking behavior, and premature discontinuation of DOAC therapy may increase stroke risk due to the loss of anticoagulation protection and potential prothrombotic rebound effects [13–16].

Evidence from large observational studies, such as GARFIELD-AF and ORBIT-AF II, has shown that oral anticoagulant (OAC) discontinuation is associated with a higher risk of all-cause mortality, stroke/systemic embolism, and myocardial infarction [16] or linked to an increased absolute risk of all-cause mortality and cardiovascular death [17]. Moreover, evidence suggests that switching between anticoagulants may further increase the risk of bleeding events [18]. Therefore, treatment continuity and patient adherence to DOACs play a central role in stroke prevention in patients with AF [6,7,18,19]. In real-world settings, 1‑year discontinuation rates for DOACs range from 22% to 40%, and nonadherence rates from 3% to 16% [20–23]. XANTUS, a prospective, observational study of patients with nonvalvular AF (NVAF), reported a rivaroxaban discontinuation rate of 20.1% after a 1-year observation period [24]. Another prospective, non-interventional registry of patients with AF treated with rivaroxaban found a discontinuation rate of 18.5% [25]. In an Italian retrospective, administrative–database study by Degli Esposti et al., patients with NVAF who received anticoagulant therapy (Vitamin K antagonists [VKAs] or DOACs) were included and stratified as naïve and non-naïve users of these agents. In this population, adherence to DOAC therapy was 76.1%. However, the design of this study limited the ability to assess the reasons for premature treatment discontinuation and evaluate the impact of several clinical factors, including comorbidities, disease severity, concurrent medications, and treatment adherence – both of which are crucial considerations in real-world clinical practice [26]. An isolated analysis of subpopulations of elderly patients receiving DOACs or VKAs showed that DOAC use was generally associated with a lower incidence of major bleeding events and better clinical outcomes than VKAs, although adherence and persistence were not assessed due to limitations in data collection [27]. These findings can be interpreted in the context of pivotal clinical trials and large real-world studies assessing rivaroxaban safety and efficacy. Pivotal randomized trials, such as the ROCKET AF, and large real-world studies, such as XANTUS, have demonstrated low rates of major bleeding and stroke in patients treated with rivaroxaban [11,24]. In ROCKET AF, patients were treated with either rivaroxaban or dose-adjusted warfarin, and no significant differences in rates of clinically significant (major and non-major) bleeding events were observed between treatment groups; however, intracranial and fatal bleeding occurred less frequently in the rivaroxaban group [11]. In XANTUS, patients receiving rivaroxaban had a low incidence of major bleeding events and stroke in routine clinical practice [24], but the study had certain limitations due to its open-label design, which introduced potential selection bias and observer bias related to knowledge of treatment allocation.

Despite the widespread use of rivaroxaban in Italy and the existence of a mandatory online therapeutic plan for DOAC prescriptions, at the time of the study initiation (December 2019), limited information was available on DOAC discontinuation or switching among patients with AF, particularly regarding the underlying reasons for treatment changes, including those involving rivaroxaban.

RITMUS-AF (Rivaroxaban Treatment Discontinuation Rates in Routine Clinical Practice in Italy in Patients with NVAF; NCT04174859) is the first prospective, multicenter, observational cohort study to investigate treatment discontinuation in patients with NVAF newly treated with rivaroxaban for the prevention of stroke and non−central nervous system systemic embolism in routine clinical practice in Italy, reflecting the real-world clinical environment in which most patients with NVAF are managed. The study also assessed the reasons for discontinuation and evaluated patient-reported adherence to rivaroxaban therapy.

## Materials and methods

### Patients

The study population comprised eligible patients with NVAF, aged ≥18 years, who were new users of rivaroxaban (either OAC-naïve or OAC-non-naïve to treatment) and who provided written informed consent before any data were collected. Patients eligible for rivaroxaban treatment solely for the prevention of stroke or systemic embolism were screened consecutively and enrolled according to the study’s inclusion and exclusion criteria [28]. Serum creatinine was measured at baseline in the majority of patients (S1 Table). Creatinine clearance (mL/min) was calculated using the Cockcroft–Gault formula, and estimated glomerular filtration rate (eGFR, mL/min/1.73 m²) was calculated using the CKD-EPI creatinine equation. Observational data suggest that approximately 15% of rivaroxaban users discontinue treatment within 12 months. Assuming that 20–50% of patients with NVAF taking rivaroxaban would discontinue it within 24 months of study entry, a sample size of 720 patients would provide a precision level between 2.9% and 3.7% (measured as the distance from the proportion to each limit of the two-sided 95% coincidence interval (CI), calculated using the normal approximation) [29]. To account for site feasibility and an expected 10% loss to follow-up, the enrollment target was set at 800 patients.

### Study design

RITMUS-AF was conducted in 31 cardiology departments across all 20 regions of Italy. The enrollment period spanned from the first patient’s first visit (FPFV) on December 10, 2019, to the last patient’s first visit (LPFV) on September 30, 2021. Final data collection was completed in June 2022. Rivaroxaban was prescribed in accordance with its marketing authorization in Italy, and all treatment decisions were made by the prescribing physician. Withdrawal from RITMUS-AF was independent of treatment decisions and did not affect participants’ medical care. Details of the study design and data collection methods have previously been described and published [28].

### Study endpoints

The primary endpoint of RITMUS-AF was the proportion of patients who discontinued rivaroxaban treatment during a maximum follow-up of 24 months. The duration of rivaroxaban treatment was defined as the time from study entry until discontinuation. Discontinuation was defined as a permanent stop of rivaroxaban treatment for more than 4 weeks, including switching to another anticoagulant. Interruptions were not considered permanent discontinuations and included: (i) temporary interruptions with restart within 4 weeks and (ii) temporary interruptions for scheduled major surgery, even if restart occurred later than 4 weeks. Secondary endpoints included: (a) the reasons for rivaroxaban discontinuation; (b) dose changes and their associated reasons; (c) switches to other therapies and their associated reasons; (d) self-reported adherence to rivaroxaban therapy, assessed using the 8-item Morisky Medication Adherence Scale (MMAS-8), as previously reported in DOAC adherence studies [30–32]; and (e) safety outcomes (adverse events of special interest [AESI], bleeding, cerebrovascular events), analyzed in the safety analysis set.

### Analysis sets

The following analysis sets were defined: (a) safety analysis set, including all enrolled patients who received at least one dose of rivaroxaban; and (b) eligible set, including all enrolled patients who received at least one dose of rivaroxaban after study entry (i.e., after signing the informed consent form) or within 15 days before signing the informed consent form, with eCRF data collection confirmed by an investigator (S1 Fig). All safety analyses were conducted using data from the safety analysis set, whereas the primary and secondary objectives were analyzed in the eligible set.

### Statistical analyses

Categorical variables were analyzed descriptively using frequency tables (absolute and relative frequencies), and continuous variables were summarized using descriptive statistics (mean, standard deviation [SD], minimum, median, quartiles, and maximum).

Patients who died, withdrew from the study, or were lost to follow-up without available treatment status information were censored at the time of the event or the last known follow-up date. The Kaplan–Meier product-limit method was used to estimate the time to rivaroxaban discontinuation, and the discontinuation rate was reported as the number of discontinuation events per 100 patient-years. The total duration of rivaroxaban therapy was calculated as the sum of days from treatment initiation to discontinuation. Kaplan–Meier estimates were used to assess treatment discontinuation over time, and discontinuation rates were reported at predefined time intervals (3, 6, 9, and 12 months) with corresponding 95% CI.

Primary and secondary endpoint results were stratified by age (<75 years vs ≥ 75 years), diabetes status (yes vs no), and prior OAC treatment (OAC-naïve vs OAC-non-naïve patients). Diabetes was selected as a stratification variable because patients with diabetes are at higher cardiovascular risk and generally represent a more clinically fragile population [7]. Data were managed by OPIS S.r.l., a contract research organization responsible for developing the electronic data capture system (including the electronic case report form [eCRF]), performing quality control, monitoring the data collection process, conducting data analyses, and transferring the dataset to Bayer. All statistical analyses were descriptive and were performed using SAS software, release 9.4 (64-bit) (SAS Institute Inc., Cary, NC, USA). Categorical variables were summarized as absolute and relative frequencies, and continuous variables as mean, standard deviation, median, quartiles, minimum and maximum. Safety outcomes were also analyzed descriptively and are presented as absolute numbers and percentages. Details of the data management and statistical analyses have previously been described and published [28].

### Ethics and consent

All study documentation received central approval and a favorable opinion from the Institutional Review Board or Ethics Committee of the coordinating site, as well as approval from the Institutional Review Board or Ethics Committee of each participating site, before local initiation. The study protocol adhered to the ethical guidelines of the 1975 Declaration of Helsinki.

## Results and discussion

Adherence to anticoagulant treatment plays a crucial role in achieving optimal therapeutic effects and improving prognosis in patients with AF. RITMUS-AF is the first prospective, observational study conducted in Italy to evaluate both the rates and the underlying reasons for DOAC treatment discontinuation in routine clinical practice.

### Baseline characteristics

A total of 821 patients were screened, of whom 805 were included in the primary endpoint analysis (S1 Fig). The eligible set consisted mostly of OAC-naïve patients (n = 599, 74.41%) without diabetes mellitus (n = 642, 79.75%), and with a mean age of 75.1 years (Table 1). These baseline characteristics are generally consistent with those reported in ROCKET AF and XANTUS. However, RITMUS-AF included a slightly higher proportion of patients aged ≥75 years and those first-diagnosed or paroxysmal AF [11,24,30]. Patients presented either symptomatic (n = 364, 45.22%) or asymptomatic (n = 441, 54.78%) forms of NVAF. Among all cases, 39.01% (n = 314) were first-diagnosed, 36.52% (n = 294) were paroxysmal, and 8.94% (n = 72) were persistent. Clinical and laboratory parameters analyzed in this study are reported in S1 Table. More than half of the patients (n = 542, 67.41%) had a CHADS_2_ (Congestive heart failure, Hypertension, Age ≥ 75 years, Diabetes mellitus, Stroke/transient ischemic attack [doubled]) score ≥2, and 757 patients (94.04%) had a CHA_2_DS_2_-VASc (Congestive heart failure, Hypertension, Age ≥ 75 years [doubled], Diabetes mellitus, Stroke/TIA/thromboembolism [doubled], Vascular disease, Age 65–74 years, Sex category [female]) score ≥2, comparable to those reported in other rivaroxaban studies [11,24]. The mean ± SD CHA_2_DS_2_-VASc score was 3.6 ± 1.48. A total of 178 patients (22.11%) had congestive heart failure, and 163 patients (20.25%) had diabetes. Seventy-three patients (9.07%) had a diathesis or history of prior bleeding events (Table 1), suggesting an expected increased risk of bleeding events following rivaroxaban treatment. However, despite this high ischemic and hemorrhagic risk, rivaroxaban treatment persistence was high, while only a small proportion of patients discontinued therapy. Patient baseline characteristics stratified by OAC-naïve status, age group, and presence of diabetes mellitus are shown in S2 Table.

**Table 1:** Patient demographic characteristics: eligible set.

Characteristic	Eligible set (N = 805)
Age (years)	
Mean (SD)	75.1 (9.80)
Median	76.0
Q1; Q3	70–82
Min; max	39–96
Age classes	
<75 years	359 (44.60%)
≥75 years	446 (55.40%)
Age at first NVAF diagnosis (years)	
N	702
Mean (SD)	72.7 (10.22)
Median	73.0
Q1; Q3	67.0–80.0
Min; max	34–96
Disease duration (months)	
N	702
Mean (SD)	22.82 (51.78)
Median	0.36
Q1; Q3	0.07–13.57
Min; max	0.03–482.69
Sex	
Male	471 (58.51%)
Female*	334 (41.49%)
Race	
White	803 (99.75%)
Other	2 (0.25%)
Current type of NVAF	
First diagnosed	314 (39.01%)
Paroxysmal	294 (36.52%)
Persistent	72 (8.94%)
Permanent	125 (15.53%)
NVAF symptoms	
Symptomatic	364 (45.22%)
Asymptomatic	441 (54.78%)
CHA_2_DS_2_-VAS_C_ score	
Mean (SD)	3.6 (1.48)
Median	4
Q1; Q3	3.0–4.0
Prior interventions for NVAF treatment^†^	114 (14.16%)
History of stroke	50 (6.21%)
Ischemic	46 (92.00%)
Hemorrhagic	3 (6.00%)
Unknown stroke	1 (2.00%)
Findings related to study indication	143 (17.76%)
Prior transient ischemic attack	34 (23.78%)
Systemic embolism	11 (7.69%)
Myocardial infarction	91 (63.64%)
Deep vein thrombosis	17 (11.89%)
Bleeding diathesis/history of prior bleeding‡	73 (9.07%)
Known coagulopathy	1 (1.37%)
Gastric ulcer	23 (31.51%)
Active cancer (diagnosis or treatment <6 months or recurrent or metastatic cancer)	18 (24.66%)
Prior intracranial bleeding	5 (6.85%)
Prior extracranial bleeding	19 (26.03%)
Other	13 (17.81%)
Risk factors for stroke or non–CNS systemic embolism	
Congestive heart failure	178 (22.11%)
Arterial hypertension	678 (84.22%)
Diabetes	163 (20.25%)
Vascular diseases	262 (32.55%)
Other risk factors	143 (17.76%)

Values are n (%) unless otherwise stated.

*Among female participants, a proportion were of reproductive age, although no pregnancies occurred during the study period. † Within the last year. ‡Percentages were computed on patients with bleeding diathesis/history of prior bleeding. Each patient could have ≥1 bleeding diathesis/history of prior bleeding conditions.

CNS: central nervous system; max: maximum; min: minimum; NVAF: nonvalvular atrial fibrillation; Q: quartile; SD: standard deviation.

### Treatment discontinuation outcomes

**Fig 1:**
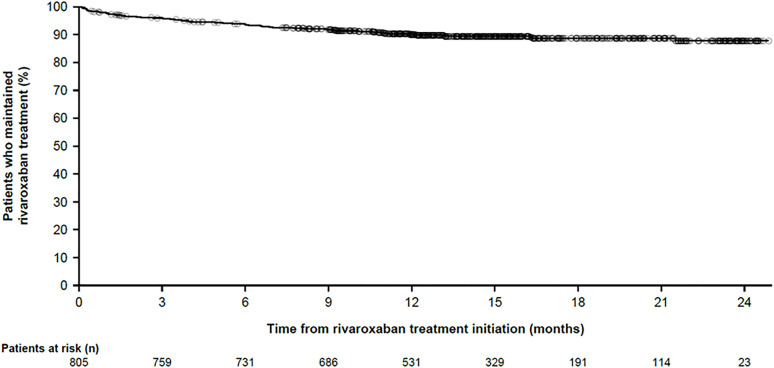
Primary endpoint. Kaplan–Meier estimated proportion of patients who maintained rivaroxaban treatment from baseline to month 24. Numbers at risk: 12 months n = 531; 24 months n = 23.

This study design enabled the collection of information not typically captured in medical claims databases or electronic medical records, such as reasons for discontinuation, dose adjustments, and patient-reported adherence. Premature treatment discontinuation occurred in 83 patients (10.31%). Among these, 74 patients (89.16%) discontinued rivaroxaban due to safety- or efficacy-related reasons, while nine patients discontinued for other reasons, including patient decision (n = 2), missing eligibility criteria (n = 1), and other causes (n = 6). The overall discontinuation rate was 8.86 per 100 patient-years (95% CI: 7.06–10.99), indicating a low treatment discontinuation rate for rivaroxaban. As shown in Fig 1, most discontinuation events occurred within the first 3 months of treatment, after which the rate plateaued. Five hundred thirty-one patients completed 12 months of follow-up, and 23 patients completed 24 months. The present study specifically examined the impact of patient age, diabetes status (presence vs absence), and OAC-naïve status on therapy discontinuation and the risk of different types of adverse events (AEs). Discontinuation rates were similar across subgroups (stratified by age, diabetes prevalence, and OAC-naïve status; S2 Fig and S3 Table), with slightly lower rates observed in patients with diabetes (7.54 per 100 patient-years [95% CI: 4.12–12.65]) compared with those without diabetes (9.19 per 100 patient-years [95% CI: 7.15–11.63]) and all other analyzed subgroups (S3 Table).

The most frequently reported reasons for discontinuation were AEs (n = 41, 49.40%) and physician decisions (i.e., treatment discontinuation based on the judgement of the treating physician; n = 25, 30.12%), while patient decisions accounted for only 8.43% (n = 7) of cases. Detailed data on specific clinical diagnoses leading to discontinuation were collected but are not presented here. Thirty patients (3.73%) in the eligible set had at least one dose adjustment, mainly due to changes in creatinine clearance (n = 23, 63.89%). One patient underwent percutaneous coronary intervention and received a reduced rivaroxaban dose for 9 days. Rivaroxaban interruptions occurred in 60 patients (7.45%), primarily due to surgical interventions (n = 27, 36.99%) or AEs (n = 28, 38.36%). Only one case of discontinuation was related to a diagnosis of cancer. Approximately half of the patients who prematurely discontinued treatment (n = 44, 53.01%) switched to another therapy. Most of them (n = 42, 95.45%) switched to other antithrombotic agents (apixaban, dabigatran, edoxaban, enoxaparin, warfarin, warfarin sodium and acetylsalicylic acid), while one patient received an agent acting on the renin–angiotensin system and one patient received a different therapeutic product. Reasons for switching included ischemic events (n = 5, 11.36%), bleedings (n = 11, 25.00%), adverse drug reactions (n = 7, 15.91%), drug-to-drug interactions (n = 1, 2.23%), and other causes (n = 20, 45.45%). Discontinuation was independent of age, diabetes status, and OAC-naïve status. Notably, most discontinuation events occurred within the first 3 months of therapy, consistent with previous reports of higher discontinuation rates in the first year of therapy compared with later years [17,26]. This early discontinuation pattern is multifactorial, involving clinical events, socioeconomic barriers, and patient-related factors. Addressing these aspects through improved patient education, financial support, and careful management of bleeding risks may help to enhance adherence to DOAC therapy and ultimately improve patient outcomes in anticoagulation management. Overall, these results align with other studies of rivaroxaban in NVAF patients [24,25].

### Self-reported adherence to rivaroxaban

**Fig 2:**
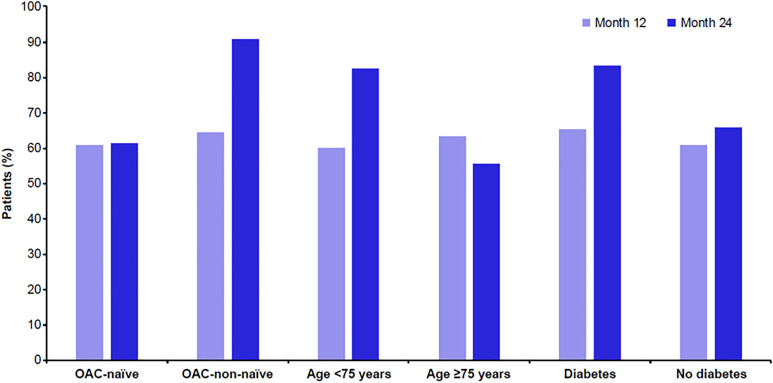
Self-reported high adherence to rivaroxaban: eligible set. Proportion of patients with high adherence based on the MMAS-8 questionnaire at 12 and 24 months. Adherence rates were calculated only in patients who attended the visits and completed the MMAS-8 questionnaire. The apparently higher proportion at 24 months reflects the smaller subgroup of patients who completed follow-up at this time point. MMAS-8: 8-item Morisky Medication Adherence Scale; OAC: oral anticoagulant.

Adherence was assessed using the MMAS-8 questionnaire, a widely used instrument that provides physicians with insights into patient behavior and potential barriers to treatment adherence [31–33]. A high proportion of patients in the eligible set reported high adherence at both 12 and 24 months. At month 12, 585 patients (72.27%) attended a site visit, and 539 of these (92.14%) completed the MMAS-8 questionnaire, with a mean ± SD MMAS-8 score of 7.49 ± 0.908. Only patients who attended the visit and completed the questionnaire were included in this analysis; patients who discontinued treatment before month 12 were not part of this dataset. Low, medium, and high adherence were reported by 44 patients (8.16%), 162 (30.06%), and 333 (61.78%), respectively. At month 24, 55 patients (6.83%) attended a final site visit, and 50 of those (90.91%) completed the MMAS-8 questionnaire, with a mean ± SD MMAS-8 score of 7.48 ± 0.972; 4 patients (8.00%), 12 (24.00%), and 34 (68.00%) reported low, medium and high adherence, respectively (S4 Table). At month 12, high adherence rates showed no substantial differences across age, diabetes status or OAC-naïve status subgroups. However, at month 24, high adherence to rivaroxaban was more frequent among patients aged <75 years compared with those aged ≥75 years (82.61% vs 55.56%), in patients with diabetes compared with those without (83.33% vs 65.91%), and in OAC-non-naïve patients at baseline than OAC-naïve (90.91% vs 61.54%; Fig 2). In our study, discontinuation appeared numerically lower among patients with diabetes; however, we did not identify specific explanatory factors. Evidence from the literature on this topic is inconsistent, with some studies reporting comparable adherence rates between patients with and without diabetes [34]. Further investigation is warranted to clarify the determinants of adherence in this subgroup. In addition, discontinuation at month 24 was lower among patients who were OAC-non-naïve at baseline. A possible explanation is that these patients were already accustomed to long-term anticoagulation and more aware of the importance of continuous therapy, having often experienced prior clinical events that reinforced treatment motivation. This observation is consistent with findings from real-world studies reporting greater persistence among patients with previous anticoagulant exposure [26,27]. Notably, only 8.43% of discontinuation cases were patient-driven, possibly reflecting the once-daily dosing regimen, which has been associated with higher adherence than twice-daily regimens [25,35]. These findings align with previously reported real-world data on DOAC adherence in Italy [26] and observations from other international cohorts [27].

### Safety outcomes

Safety outcomes, analyzed in the safety analysis set, are summarized in Table 2. A total of 176 patients (21.70%) experienced AEs, of whom 88 (10.85%) had serious AEs and 48 (5.92%) with suspected drug-related AEs. AESI occurred in 52 patients (6.41%), most commonly bleeding events (n = 46, 5.67%). Life-threatening major bleeding occurred in <0.5% of patients, including one case of intracranial hemorrhage. Only one case of ischemic stroke (0.12%) was reported. During the study, 36 deaths occurred (4.44%), but only 2 of those were considered related to rivaroxaban treatment. Overall, rivaroxaban therapy was associated with a favorable benefit–risk profile in this high-risk population in routine clinical practice in Italy, although the interpretation of these findings is limited by the study design and sample size. These findings are consistent with those from pivotal clinical trials of rivaroxaban in NVAF patients, including ROCKET AF [11,30], and from large real-world studies, such as XANTUS [24], which also demonstrated low rates of bleeding events in patients treated with rivaroxaban.

**Table 2:** Patients with AEs: Safety analysis set.

Type of AE	Safety analysis set (N = 811), n (%)
Any AEs	176 (21.70%)
SAEs	88 (10.85%)
Suspected drug-related AEs	48 (5.92%)
Cerebral hemorrhage	1 (0.12%)
Intracranial hemorrhage	1 (0.12%)
Ischemic stroke	1 (0.12%)
Subarachnoid hemorrhage (traumatic)	2 (0.25%)
Gastrointestinal hemorrhage	2 (0.25%)
AESI	52 (6.41%)
Bleeding events	46 (5.67%)
Bone marrow toxicity	0 (0.00%)
Thrombocytopenia	1 (0.12%)
Liver-related events	1 (0.12%)
Anaphylaxis	0 (0.00%)
Cutaneous events	1 (0.12%)
Skin vasculitis	0 (0.00%)
Renal failure	3 (0.37%)
Pregnancy	0 (0.00%)
AEs leading to temporary interruption of treatment	30 (3.70%)
AEs leading to discontinuation of treatment	43 (5.30%)
AEs with a fatal outcome	36 (4.44%)

The table shows the number of patients from the RITMUS-AF safety analysis set for whom the listed AEs were reported. Each patient could experience more than one AE. A total of 256 AEs were reported in the RITMUS-AF safety analysis set.

AE: adverse event; AESI: adverse event of special interest; RITMUS-AF: Rivaroxaban Treatment Discontinuation Rates in Routine Clinical Practice in Italy in Patients with Nonvalvular Atrial Fibrillation; SAE: serious adverse event.

### Strengths of RITMUS-AF

The present study has several strengths. Its prospective design enabled the capture of more comprehensive information on rivaroxaban treatment discontinuation rates than non-prospective studies, which are often limited by the parameters recorded in the source data. The inclusion of 31 centers across all 20 Italian regions ensured that the findings are representative of nationwide routine clinical practice and, although the study was conducted in a single country, the results are consistent with those from other developed countries [6,16,17]. Data collection followed the standards of routine clinical care, supporting the generalizability of the findings to the broader European patient population. Another strength of this study is the assessment of the reasons for rivaroxaban discontinuation**─**a factor often underreported in similar studies but relevant to clinical decision-making. Understanding these reasons may help clinicians optimize patient care. The follow-up period of up to two years provided valuable insights into temporal patterns of discontinuation. In Italy, all prescribed treatments are recorded by law in a web-based therapeutic plan, ensuring that DOACs are prescribed in accordance with international guideline recommendations and the summary of product characteristics. Moreover, all treatment-related AEs are reported to the Italian Medicines Agency (AIFA) [36].

### Limitations of RITMUS-AF

A total of 33 patients were lost to follow-up, resulting in missing treatment status for these cases. However, all patients who were lost to follow-up or withdrew were censored at the time of their last available data point, as described in the Methods (Kaplan–Meier product-limit method). This approach ensured that the data were appropriately analyzed, although the possibility of residual bias due to missing data cannot be completely excluded. Although the Kaplan–Meier method accounts for variable follow-up times, the reduced number of patients at later time points limits the precision of the discontinuation estimates. Furthermore, patients who completed 24 months of follow-up are likely to represent a subgroup with fewer clinical events and inherently higher adherence, which may have introduced selection bias. Historical medical data were not always available, and variations in treatment practices or reporting across centers could have introduced bias. Adherence data were collected through patient interviews, which may be subject to recall bias; however, a widely used standardized questionnaire (MMAS-8) was used to mitigate this risk. Moreover, all analyses were descriptive by design, and no inferential statistics were performed; as such, the findings should be interpreted as descriptive observations, without formal hypothesis testing. Finally, the study was conducted during the COVID-19 pandemic, which may have limited patient enrollment and affected study execution.

## Conclusion

RITMUS-AF found that premature rivaroxaban discontinuation was low, whereas patient-reported adherence was high among patients with NVAF in routine clinical practice in Italy. This study addresses the scarcity of real-world data on rivaroxaban treatment discontinuation and provides valuable insights into the management of patients receiving rivaroxaban for stroke prevention.

Future studies could evaluate potential predictors of treatment discontinuation in clinical practice, investigate causal relationships underlying discontinuation trends, and explore additional patient subgroups.

Despite its limitations, the study focuses on patient age, comorbidities (such as diabetes), and OAC-naïve status, providing important insights into treatment discontinuation patterns and AE incidence. The low treatment discontinuation rate of rivaroxaban observed in this real-world population at high ischemic and hemorrhagic risk is clinically reassuring, given the low proportion of patients who discontinued treatment. Overall, these findings enhance the understanding of the benefit–risk balance of DOAC therapy and may help inform clinical decision-making regarding its use in routine practice.

## Supporting information

S1 Table Baseline clinical and laboratory parameters: eligible set (N = 805). Some patients underwent partial evaluation (i.e., not all parameters were collected). Creatinine clearance (mL/min) and eGFR (mL/min/1.73 m2) were derived from serum creatinine (μmol/L) using the Cockcroft–Gault formula and CKD-EPI creatinine equation formula, respectively.
(DOCX)

S2 Table Patient demographic characteristics by subgroup: eligible set (N = 805). (DOCX)

S3 Table Rate of treatment discontinuation according to stratification by OAC-naïve status, age, and diabetes prevalence. (DOCX)

S4 Table Self-reported adherence to rivaroxaban therapy according to MMAS-8: eligible set. Results based on the MMAS-8 at 12 and 24 months.
(DOCX)

S1 Fig Study population. Enrolled set (n = 812): all screened patients who were considered eligible to be enrolled in the study. Safety analysis set (n = 811): all enrolled patients who took ≥1 dose of rivaroxaban. Eligible set (n = 805): all enrolled patients who took ≥1 dose of rivaroxaban after the study entry with eCRF-collected data confirmed by an investigator. Seven patients were excluded from the eligible set; * 1 patient met > 1 exclusion criteria and was therefore counted more than once in category totals but excluded only once from the eligible set. All safety analyses were conducted using data from the safety analysis set, while primary and secondary objectives were analyzed in the eligible set. eCRF: electronic case report form.
(PNG)

S2 Fig Primary endpoint stratified by subgroups. Kaplan–Meier estimates of treatment discontinuation stratified by (A) diabetes status, (B) OAC-naïve status, and (C) age. OAC: oral anticoagulant.
(PNG)

## References

[1] Ball J, Carrington MJ, McMurray JJV, Stewart S. Atrial fibrillation: profile and burden of an evolving epidemic in the 21st century. Int J Cardiol. 2013;167(5):1807–24. doi: 10.1016/j.ijcard.2012.12.093 23380698

[2] Chugh SS, Havmoeller R, Narayanan K, Singh D, Rienstra M, Benjamin EJ, et al. Worldwide epidemiology of atrial fibrillation: a Global Burden of Disease 2010 Study. Circulation. 2014;129(8):837–47. doi: 10.1161/CIRCULATIONAHA.113.005119 24345399 PMC4151302

[3] Di Carlo A, Bellino L, Consoli D, Mori F, Zaninelli A, Baldereschi M, et al. Prevalence of atrial fibrillation in the Italian elderly population and projections from 2020 to 2060 for Italy and the European Union: the FAI Project. Europace. 2019;21(10):1468–75. doi: 10.1093/europace/euz141 31131389

[4] Di Carlo A, Zaninelli A, Mori F, Consoli D, Bellino L, Baldereschi M, et al. Prevalence of Atrial Fibrillation Subtypes in Italy and Projections to 2060 for Italy and Europe. J Am Geriatr Soc. 2020;68(11):2534–41. doi: 10.1111/jgs.16748 32786082

[5] Molteni M, Polo Friz H, Primitz L, Marano G, Boracchi P, Cimminiello C. The definition of valvular and non-valvular atrial fibrillation: results of a physicians’ survey. Europace. 2014;16(12):1720–5. doi: 10.1093/europace/euu178 25087153

[6] Odutayo A, Wong CX, Hsiao AJ, Hopewell S, Altman DG, Emdin CA. Atrial fibrillation and risks of cardiovascular disease, renal disease, and death: systematic review and meta-analysis. BMJ. 2016;354:i4482. doi: 10.1136/bmj.i4482 27599725

[7] Van Gelder IC, Rienstra M, Bunting KV, Casado-Arroyo R, Caso V, Crijns HJGM, et al. 2024 ESC Guidelines for the management of atrial fibrillation developed in collaboration with the European Association for Cardio-Thoracic Surgery (EACTS). Eur Heart J. 2024;45(36):3314–414. doi: 10.1093/eurheartj/ehae176 39210723

[8] Connolly SJ, Ezekowitz MD, Yusuf S, Eikelboom J, Oldgren J, Parekh A, et al. Dabigatran versus warfarin in patients with atrial fibrillation. N Engl J Med. 2009;361(12):1139–51. doi: 10.1056/NEJMoa0905561 19717844

[9] Giugliano RP, Ruff CT, Braunwald E, Murphy SA, Wiviott SD, Halperin JL, et al. Edoxaban versus warfarin in patients with atrial fibrillation. N Engl J Med. 2013;369(22):2093–104. doi: 10.1056/NEJMoa1310907 24251359

[10] Granger CB, Alexander JH, McMurray JJV, Lopes RD, Hylek EM, Hanna M, et al. Apixaban versus warfarin in patients with atrial fibrillation. N Engl J Med. 2011;365(11):981–92. doi: 10.1056/NEJMoa1107039 21870978

[11] Patel MR, Mahaffey KW, Garg J, Pan G, Singer DE, Hacke W, et al. Rivaroxaban versus warfarin in nonvalvular atrial fibrillation. N Engl J Med. 2011;365(10):883–91. doi: 10.1056/NEJMoa1009638 21830957

[12] Brown MT, Bussell JK. Medication adherence: WHO cares? Mayo Clin Proc. 2011;86(4):304–14. doi: 10.4065/mcp.2010.0575 21389250 PMC3068890

[13] Rivera-Caravaca JM, Roldán V, Esteve-Pastor MA, Valdés M, Vicente V, Lip GYH, et al. Cessation of oral anticoagulation is an important risk factor for stroke and mortality in atrial fibrillation patients. Thromb Haemost. 2017;117(7):1448–54. doi: 10.1160/TH16-12-0961 28331926

[14] Kim YD, Lee JH, Jung YH, Cha M-J, Choi HY, Nam CM, et al. Effect of warfarin withdrawal on thrombolytic treatment in patients with ischaemic stroke. Eur J Neurol. 2011;18(9):1165–70. doi: 10.1111/j.1468-1331.2011.03363.x 21314856

[15] Genewein U, Haeberli A, Straub PW, Beer JH. Rebound after cessation of oral anticoagulant therapy: the biochemical evidence. Br J Haematol. 1996;92(2):479–85. doi: 10.1046/j.1365-2141.1996.d01-1499.x 8603020

[16] Cools F, Johnson D, Camm AJ, Bassand J-P, Verheugt FWA, Yang S, et al. Risks associated with discontinuation of oral anticoagulation in newly diagnosed patients with atrial fibrillation: results from the GARFIELD-AF Registry. J Thromb Haemost. 2021;19(9):2322–34. doi: 10.1111/jth.15415 34060704 PMC8390436

[17] Jackson LR 2nd, Kim S, Blanco R, Thomas L, Ansell J, Fonarow GC, et al. Discontinuation rates of warfarin versus direct acting oral anticoagulants in US clinical practice: results from Outcomes Registry for Better Informed Treatment of Atrial Fibrillation II (ORBIT-AF II). Am Heart J. 2020;226:85–93. doi: 10.1016/j.ahj.2020.04.016 32526533

[18] Steffel J, Collins R, Antz M, Cornu P, Desteghe L, Haeusler KG, et al. 2021 European Heart Rhythm Association practical guide on the use of non-vitamin K antagonist oral anticoagulants in patients with atrial fibrillation. Europace. 2021;23(10):1612–76. doi: 10.1093/europace/euab065 33895845 PMC11636576

[19] Zalesak M, Siu K, Francis K, Yu C, Alvrtsyan H, Rao Y, et al. Higher persistence in newly diagnosed nonvalvular atrial fibrillation patients treated with dabigatran versus warfarin. Circ Cardiovasc Qual Outcomes. 2013;6(5):567–74. doi: 10.1161/CIRCOUTCOMES.113.000192 23922182

[20] Banerjee A, Benedetto V, Gichuru P, Burnell J, Antoniou S, Schilling RJ, et al. Adherence and persistence to direct oral anticoagulants in atrial fibrillation: a population-based study. Heart. 2020;106(2):119–26. doi: 10.1136/heartjnl-2019-315307 31601729 PMC6993026

[21] Forslund T, Wettermark B, Hjemdahl P. Comparison of treatment persistence with different oral anticoagulants in patients with atrial fibrillation. Eur J Clin Pharmacol. 2016;72(3):329–38. doi: 10.1007/s00228-015-1983-z 26613954

[22] Gorst-Rasmussen A, Skjøth F, Larsen TB, Rasmussen LH, Lip GYH, Lane DA. Dabigatran adherence in atrial fibrillation patients during the first year after diagnosis: a nationwide cohort study. J Thromb Haemost. 2015;13(4):495–504. doi: 10.1111/jth.12845 25594442

[23] Ruigómez A, Vora P, Balabanova Y, Brobert G, Roberts L, Fatoba S, et al. Discontinuation of non-Vitamin K antagonist oral anticoagulants in patients with non-valvular atrial fibrillation: a population-based cohort study using primary care data from The Health Improvement Network in the UK. BMJ Open. 2019;9(10):e031342. doi: 10.1136/bmjopen-2019-031342 31630107 PMC6803078

[24] Camm AJ, Amarenco P, Haas S, Hess S, Kirchhof P, Kuhls S, et al. XANTUS: a real-world, prospective, observational study of patients treated with rivaroxaban for stroke prevention in atrial fibrillation. Eur Heart J. 2016;37(14):1145–53. doi: 10.1093/eurheartj/ehv466 26330425 PMC4823634

[25] Beyer-Westendorf J, Förster K, Ebertz F, Gelbricht V, Schreier T, Göbelt M, et al. Drug persistence with rivaroxaban therapy in atrial fibrillation patients-results from the Dresden non-interventional oral anticoagulation registry. Europace. 2015;17(4):530–8. doi: 10.1093/europace/euu319 25694537 PMC4381834

[26] Degli Esposti L, Andretta M, Di Pasquale G, Gambera M, Saragoni S, Perrone V, et al. Clinical characteristics and health care resources in patients treated with oral anticoagulants: evidences from italian administrative databases. Vasc Health Risk Manag. 2019;15:429–37. doi: 10.2147/VHRM.S216749 31632047 PMC6793461

[27] Patti G, Pecen L, Lucerna M, Huber K, Rohla M, Renda G, et al. Net clinical benefit of non-vitamin K antagonist vs vitamin K antagonist anticoagulants in elderly patients with atrial fibrillation. Am J Med. 2019;132(6):749-757.e5. doi: 10.1016/j.amjmed.2018.12.036 30664837

[28] Piero Perna G, Vora P, Gandini E, Francesca Lusona C, Tosarello D. Persistence to rivaroxaban therapy for stroke prevention in clinical practice in Italy: Rationale and design of the RITMUS-AF prospective observational cohort study. Int J Cardiol Heart Vasc. 2023;47:101229. doi: 10.1016/j.ijcha.2023.101229 37292062 PMC10244690

[29] Vedovati MC, Verdecchia P, Giustozzi M, Molini G, Conti S, Pierpaoli L, et al. Permanent discontinuation of non vitamin K oral anticoagulants in real life patients with non-valvular atrial fibrillation. Int J Cardiol. 2017;236:363–9. doi: 10.1016/j.ijcard.2017.01.098 28131705

[30] Halperin JL, Hankey GJ, Wojdyla DM, Piccini JP, Lokhnygina Y, Patel MR, et al. Efficacy and safety of rivaroxaban compared with warfarin among elderly patients with nonvalvular atrial fibrillation in the Rivaroxaban Once Daily, Oral, Direct Factor Xa Inhibition Compared With Vitamin K Antagonism for Prevention of Stroke and Embolism Trial in Atrial Fibrillation (ROCKET AF). Circulation. 2014;130(2):138–46. doi: 10.1161/CIRCULATIONAHA.113.005008 24895454

[31] Hayat A, Själander A, Wallvik J. Direct oral anticoagulants: patient reported adherence and minor bleedings. J Thromb Thrombolysis. 2023;56(1):55–64. doi: 10.1007/s11239-023-02797-8 37119356 PMC10284977

[32] Di Gennaro L, Monaco M, Riccio C, De Candia E, Alberelli MA, di Martino C, et al. Direct oral anticoagulants and therapeutic adherence: do not let your guard down. Acta Cardiol. 2022;77(3):243–9. doi: 10.1080/00015385.2021.1908702 33896375

[33] Patel SI, Cherington C, Scherber R, Barr K, McLemore R, Morisky DE, et al. Assessment of patient adherence to direct oral anticoagulant vs warfarin therapy. JOM. 2017;117(1):7–15. doi: 10.7556/jaoa.2017.002 28055097

[34] Bonsu KO, Young SW, Lee T, Nguyen HV, Chitsike RS. Self-reported adherence to direct oral anticoagulants versus warfarin therapy in a specialized thrombosis service-a cross-sectional study of patients in a Canadian Health Region. Eur J Clin Pharmacol. 2023;79(1):117–25. doi: 10.1007/s00228-022-03418-8 36399203

[35] Nelson WW, Song X, Thomson E, Smith DM, Coleman CI, Damaraju CV, et al. Medication persistence and discontinuation of rivaroxaban and dabigatran etexilate among patients with non-valvular atrial fibrillation. Curr Med Res Opin. 2015;31(10):1831–40. doi: 10.1185/03007995.2015.1074064 26211816

[36] Piccinni C, Addesi A, Pedrini A, Esposito I, Rampazzo R, Mezzalira L, et al. Piani terapeutici dei farmaci: quanti e quali? fotografia di uno strumento di appropriatezza prescrittiva e assistenziale. Recenti Prog Med. 2021. doi: 10.1701/3584.35683 33877085

