# Supplementary material for: Rivaroxaban treatment discontinuation rates in patients with nonvalvular atrial fibrillation in Italian clinical practice: RITMUS-AF
Source: PLoS One. 2026 Feb 12;21(2):e0341633. doi: 10.1371/journal.pone.0341633 (PMC12900358; doi:10.1371/journal.pone.0341633)
Supplement: S1 Table — Some patients underwent partial evaluation (i.e., not all parameters were collected). Creatinine clearance (mL/min) and eGFR (mL/min/1.73 m2) were derived from serum creatinine (μmol/L) using the Cockcroft–Gault formula and CKD-EPI creatinine equation formula, respectively. (DOCX) [file pone.0341633.s001.docx]

**S1 Table. Baseline clinical and laboratory parameters: Eligible Set (N=805).**

| **Parameter** | **n (%)** | **Mean (SD)** | **Median** | **Q1–Q3** | **Min–Max** |
| --- | --- | --- | --- | --- | --- |
| Height (cm) | 733 (91.06%) | 168.1 (9.23) | 169 | 160.0–175.0 | 145–197 |
| Weight (kg) | 775 (96.27%) | 77.8 (15.90) | 76 | 67.0–86.0 | 40–150 |
| BMI (kg/m^2^) | 733 (91.06%) | 27.4 (4.84) | 26.8 | 24.3–29.8 | 16–51 |
| Vital signs | | | | | |
| Any vital signs measured | 795 (98.76%) | – | – | – | – |
| SBP (mmHg) | 787 (97.76%) | 130.0 (14.80) | 130 | 120.0–140.0 | 90–200 |
| DBP (mmHg) | 787 (97.76%) | 77.2 (9.35) | 80 | 70.0–80.0 | 50–120 |
| Heart rate (beats/min) | 767 (95.28%) | 78.4 (19.81) | 73 | 65.0–86.0 | 42–160 |
| Laboratory parameters | | | | | |
| Any laboratory tests performed | 779 (96.77%) | – | – | – | – |
| Hemoglobin (g/L) | 739 (91.80%) | 135.1 (17.92) | 137.0 | 123.0–148.0 | 76–189 |
| Serum creatinine (μmol/L) | 755 (93.79%) | 88.3 (25.87) | 82.2 | 70.7–100.8 | 40–211 |
| Creatinine clearance (mL/min) | 725 (90.06%) | 71.0 (29.22) | 65.8 | 50.9–83.9 | 19–240 |
| eGFR (mL/min/1.73 m^2^) | 755 (93.79%) | 69.2 (18.43) | 70.2 | 55.2–83.9 | 24–115 |

Some patients underwent partial evaluation (i.e., not all parameters were collected). Creatinine clearance (mL/min) and eGFR (mL/min/1.73 m2) were derived from serum creatinine (μmol/L) using the Cockcroft–Gault formula and CKD-EPI creatinine equation formula, respectively.

BMI: body mass index; CKD-EPI: Chronic Kidney Disease Epidemiology Collaboration; DBP: diastolic blood pressure; eGFR: estimated glomerular filtration rate; max: maximum; min: minimum; Q: quartile; SBP: systolic blood pressure; SD: standard deviation.
