# Supplementary material for: Rivaroxaban treatment discontinuation rates in patients with nonvalvular atrial fibrillation in Italian clinical practice: RITMUS-AF
Source: PLoS One. 2026 Feb 12;21(2):e0341633. doi: 10.1371/journal.pone.0341633 (PMC12900358; doi:10.1371/journal.pone.0341633)
Supplement: S2 Table — (DOCX) [file pone.0341633.s002.docx]

**S2 Table.** **Patient demographic characteristics by subgroup: Eligible Set (N=805).**

| **Patient Demographic Characteristics** | **OAC-naïve status** | | | **Age class** | | **Presence of diabetes** | |
| --- | --- | --- | --- | --- | --- | --- | --- |
|  | **Non-naïve (n=206)** | **Naïve (n=599)** | | **<75 years (n=359)** | **≥75 years (n=446)** | **No (n=642)** | **Yes (n=163)** |
| Age (years) | | | | | | | |
| Mean (SD) | 77.7 (8.89) | 74.2 (9.95) | | 66.5 (7.19) | 82.1 (4.84) | 75.0 (9.95) | 75.4 (9.23) |
| Median | 78.0 | 75.0 | | 68.0 | 82.0 | 76.0 | 76.0 |
| Q1–Q3 | 73–84 | 69–81 | 63–72 | | 78–85 | 69–82 | 71–82 |
| Min–max | 42–95 | 39–96 | 39–74 | | 75–96 | 42–95 | 39–96 |
| Age class | | | | | | | |
| <75 years | 61 (29.61%) | 298 (49.75%) | 359 (100.0%) | | 0 | 281 (43.77%) | 78 (47.85%) |
| ≥75 years | 145 (70.39%) | 301 (50.25%) | 0 | | 446 (100.0%) | 361 (56.23%) | 85 (52.15%) |
| Age at first NVAF diagnosis (years) | | | | | | | |
| n | 160 | 542 | 331 | | 371 | 565 | 137 |
| Mean (SD) | 71.4 (10.00) | 73.1 (10.26) | 65.1 (7.86) | | 79.4 (6.83) | 72.3 (10.35) | 74.0 (9.58) |
| Median | 71.0 | 74.0 | 67.0 | | 79.0 | 73.0 | 74.0 |
| Q1–Q3 | 67.0–78.0 | 67.0–80.0 | 61.0–72.0 | | 76.0–84.0 | 66.0–80.0 | 70.0–80.0 |
| Min–max | 39–95 | 34–96 | 34–74 | | 39–96 | 34–95 | 39–96 |
| Disease duration (months) | | | | | | | |
| n | 160 | 542 | 331 | | 371 | 565 | 137 |
| Mean (SD) | 69.69 (74.41) | 8.99 (31.70) | 15.84 (40.73) | | 29.05 (59.30) | 23.86 (52.45) | 18.54 (48.86) |
| Median | 46.03 | 0.20 | 0.26 | | 0.46 | 0.43 | 0.26 |
| Q1–Q3 | 13.19–97.70 | 0.03–0.95 | 0.07–4.80 | | 0.03–29.76 | 0.07–15.50 | 0.03–5.42 |
| Min–max | 0.03–482.69 | 0.03–247.46 | 0.03–247.46 | | 0.03–482.69 | 0.03–482.69 | 0.03–285.42 |
| Sex | | | | | | | |
| Male | 131 (63.59%) | 340 (56.76%) | 246 (68.52%) | | 225 (50.45%) | 366 (57.01%) | 105 (64.42%) |
| Female | 75 (36.41%) | 259 (43.24%) | 113 (31.48%) | | 221 (49.55%) | 276 (42.99%) | 58 (35.58%) |
| Race | | | | | | | |
| White | 204 (99.03%) | 599 (100.0%) | 358 (99.72%) | | 445 (99.78%) | 640 (99.69%) | 163 (100.0%) |
| Other | 2 (0.97%) | 0 | 1 (0.28%) | | 1 (0.22%) | 2 (0.31%) | 0 |
| Current type of NVAF | | | | | | | |
| First diagnosed | 10 (4.85%) | 304 (50.75%) | 149 (41.50%) | | 165 (37.00%) | 254 (39.56%) | 60 (36.81%) |
| Paroxysmal | 79 (38.35%) | 215 (35.89%) | 145 (40.39%) | | 149 (33.41%) | 233 (36.29%) | 61 (37.42%) |
| Persistent | 25 (12.14%) | 47 (7.85%) | 37 (10.31%) | | 35 (7.85%) | 55 (8.57%) | 17 (10.43%) |
| Permanent | 92 (44.66%) | 33 (5.51%) | 28 (7.80%) | | 97 (21.75%) | 100 (15.58%) | 25 (15.34%) |
| NVAF symptoms | | | | | | | |
| Symptomatic | 54 (26.21%) | 310 (51.75%) | 195 (54.32%) | | 169 (37.89%) | 302 (47.04%) | 62 (38.04%) |
| Asymptomatic | 152 (73.79%) | 289 (48.25%) | 164 (45.68%) | | 277 (62.11%) | 340 (52.96%) | 101 (61.96%) |
| Prior interventions for NVAF treatment* | 34 (16.50%) | 80 (13.36%) | 67 (18.66%) | | 47 (10.54%) | 95 (14.80%) | 19 (11.66%) |
| History of stroke | 11 (5.34%) | 39 (6.51%) | 18 (5.01%) | | 32 (7.17%) | 36 (5.61%) | 14 (8.59%) |
| Ischemic | 9 (81.82%) | 37 (94.87%) | 16 (88.89%) | | 30 (93.75%) | 33 (91.67%) | 13 (92.86%) |
| Hemorrhagic | 2 (18.18%) | 1 (2.56%) | 2 (11.11%) | | 1 (3.13%) | 2 (5.56%) | 1 (7.14%) |
| Unknown stroke | 0 | 1 (2.56%) | 0 | | 1 (3.13%) | 1 (2.78%) | 0 |
| Findings related to study indication | 42 (20.39%) | 101 (16.86%) | 61 (16.99%) | | 82 (18.39%) | 109 (16.98%) | 34 (20.86%) |
| Prior transient ischemic attack | 11 (26.19%) | 23 (22.77%) | 13 (21.31%) | | 21 (25.61%) | 28 (25.69%) | 6 (17.65%) |
| Systemic embolism | 3 (7.14%) | 8 (7.92%) | 3 (4.92%) | | 8 (9.76%) | 9 (8.26%) | 2 (5.88%) |
| Myocardial infarction | 24 (57.14%) | 67 (66.34%) | 43 (70.49%) | | 48 (58.54%) | 67 (61.47%) | 24 (70.59%) |
| Deep vein thrombosis | 6 (14.29%) | 11 (10.89%) | 6 (9.84%) | | 11 (13.41%) | 13 (11.93%) | 4 (11.76%) |

Values are n (%) unless otherwise stated.

*Within the last year.

Max: maximum; Min: minimum; NVAF: nonvalvular atrial fibrillation; OAC: oral anticoagulant; Q: quartile; SD: standard deviation.
