# Supplementary material for: Rivaroxaban treatment discontinuation rates in patients with nonvalvular atrial fibrillation in Italian clinical practice: RITMUS-AF
Source: PLoS One. 2026 Feb 12;21(2):e0341633. doi: 10.1371/journal.pone.0341633 (PMC12900358; doi:10.1371/journal.pone.0341633)
Supplement: S3 Table — (DOCX) [file pone.0341633.s003.docx]

**S3 Table.** **Rate of treatment discontinuation according to stratification by OAC-naïve status, age, and diabetes prevalence.**

|  | **Eligible Set** | **OAC-naïve status** | | **Age** | | **Presence of diabetes** | |
| --- | --- | --- | --- | --- | --- | --- | --- |
|  | **Total (N=805)** | **Non-naïve (N=206)** | **Naïve (N=599)** | **<75 years (N=359)** | **≥75 years (N=446)** | **No**  **(N=642)** | **Yes (N=163)** |
| Rate of discontinuation per 100 patient-years (95% CI) | 8.86 (7.06–10.99) | 8.76 (5.42–13.39) | 8.90 (6.82–11.41) | 9.07 (6.42–12.45) | 8.69 (6.34–11.63) | 9.19 (7.15–11.63) | 7.54 (4.12–12.65) |

CI: confidence interval; OAC: oral anticoagulant.
