# Supplementary material for: Rivaroxaban treatment discontinuation rates in patients with nonvalvular atrial fibrillation in Italian clinical practice: RITMUS-AF
Source: PLoS One. 2026 Feb 12;21(2):e0341633. doi: 10.1371/journal.pone.0341633 (PMC12900358; doi:10.1371/journal.pone.0341633)
Supplement: S4 Table — Results based on the MMAS-8 at 12 and 24 months. (DOCX) [file pone.0341633.s004.docx]

**S4 Table.** **Self-reported adherence to rivaroxaban therapy according to MMAS-8: Eligible Set.**

|  | **Eligible Set (N=805), n (%)** | |
| --- | --- | --- |
|  | **Site visit – Month 12** | **Final visit – Month 24** |
| Patients performing the visit | 585 (72.67%) | 55 (6.83%) |
| MMAS-8 administered* | 539 (92.14%) | 50 (90.91%) |
| MMAS-8 total score^†^ | | |
| n | 539 | 50 |
| Mean (SD) | 7.49 (0.908) | 7.48 (0.972) |
| Median | 8 | 8 |
| Q1–Q3 | 7.00–8.00 | 7.00–8.00 |
| Min–max | 1.5–8.0 | 4.5–8.0 |
| MMAS-8 adherence class^†^ | | |
| Low | 44 (8.16%) | 4 (8.00%) |
| Medium | 162 (30.06%) | 12 (24.00%) |
| High | 333 (61.78%) | 34 (68.00%) |

Results based on the MMAS-8 at 12 and 24 months.

* Percentages were calculated on patients performing the visit. ^†^ Total scores were calculated on patients with MMAS-8 administered and only for completed questionnaires.

Max: maximum; Min: minimum; MMAS-8: 8-item Morisky Medication Adherence Scale; Q: quartile; SD: standard deviation.
