# Supplementary figures and images for: Rivaroxaban treatment discontinuation rates in patients with nonvalvular atrial fibrillation in Italian clinical practice: RITMUS-AF
Source: PLoS One. 2026 Feb 12;21(2):e0341633. doi: 10.1371/journal.pone.0341633 (PMC12900358; doi:10.1371/journal.pone.0341633)

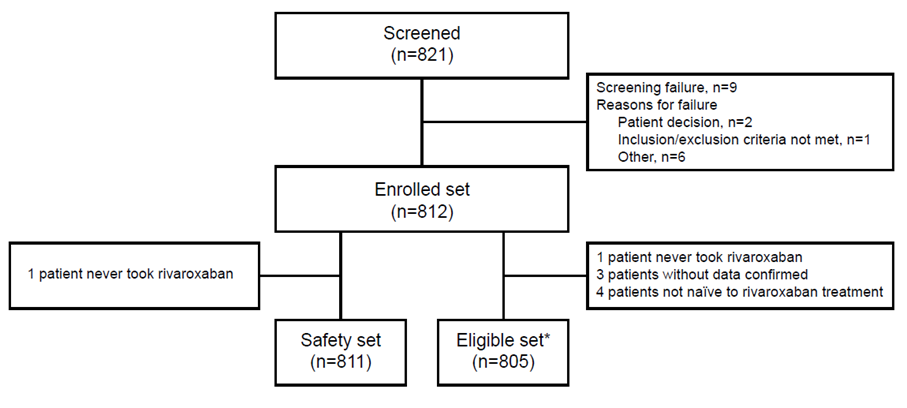

Supplement: S1 Fig — Enrolled set (n = 812): all screened patients who were considered eligible to be enrolled in the study. Safety analysis set (n = 811): all enrolled patients who took ≥1 dose of rivaroxaban. Eligible set (n = 805): all enrolled patients who took ≥1 dose of rivaroxaban after the study entry with eCRF-collected data confirmed by an investigator. Seven patients were excluded from the eligible set; * 1 patient met > 1 exclusion criteria and was therefore counted more than once in category totals but excluded only once from the eligible set. All safety analyses were conducted using data from the safety analysis set, while primary and secondary objectives were analyzed in the eligible set. eCRF: electronic case report form. (PNG) [file pone.0341633.s005.png]

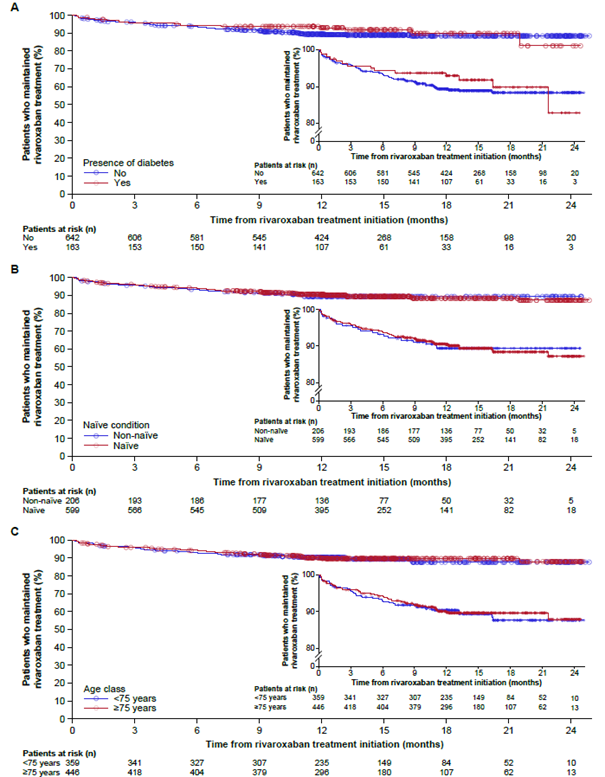

Supplement: S2 Fig — Kaplan–Meier estimates of treatment discontinuation stratified by (A) diabetes status, (B) OAC-naïve status, and (C) age. OAC: oral anticoagulant. (PNG) [file pone.0341633.s006.png]
